# The complete chloroplast genomes of *Trollius farreri* and *Anemone taipaiensis* (Ranunculaceae)

**DOI:** 10.1080/23802359.2019.1671248

**Published:** 2019-09-27

**Authors:** Ben Yu, Ruiting Zhang, Qian Yang, Bei Xu, Zhan-Lin Liu

**Affiliations:** Key Laboratory of Resource Biology and Biotechnology in Western China (Ministry of Education), College of Life Sciences, Northwest University, Xi’an, China

**Keywords:** *Trollius farreri*, *Anemone taipaiensis*, Ranunculaceae, chloroplast genome, phylogeny

## Abstract

Ranunculaceae, with high morphological biodiversity and widely ecological amplitude tolerance, has been considered as a new good model for adaptive evolution study. In this study, two high-quality chloroplast genomes of Ranunculaceae were determined by next-generation sequencing technologies. The plastomes of *Trollius farreri* and *Anemone taipaiensis* exhibit a conserved quadripartite structure, with 160,612 bp and 160,214 bp in length, presenting similar GC contents (38% and 37.6%). The chloroplast genome of *Trollius farreri* contains 131 genes, including 87 protein-coding genes, 8 rRNA, and 36 tRNA genes, while the plastome of *Anemone taipaiensis* harbours 137 genes, including 93 protein-coding genes, 8 rRNA, and 36 tRNA genes. Phylogenetic tree constructed with 21 plastomes of Ranunculaceae species indicates that *Trollius farreri* and *Anemone taipaiensis* are closely related to their congeneric species, respectively. The phylogenetic relationships are inconsistent with the current classification of Ranunculaceae.

Ranunculaceae is a basal eudicotyledonous family including over 2000 species with a global distribution. For its diversified morphological traits and widely ecological tolerance, Ranunculaceae has been considered as a newly model for evo-devo and evolutionary adaptation studies (Zhai et al. 2019). The infra-familial relationships in Ranunculaceae are still controversial, partly ascribed to the limited genomic data used. The genus *Trollius* has a large R type chromosome, which is considered as the most primitive group of Ranunculaceae. *Trollius farreri*, a perennial herb belonging to Adonideae tribe, is used as traditional Chinese medicine with antiviral activity. *Anemone taipaiensis*, an endemic species in Qinling Mountains, China, is traditionally used to treat rheumatism and phlebitis, and also has significant anti-tumour activity as recently reported (Li et al. 2013). In this study, we determined the complete chloroplast genome sequences of *Trollius farreri* and *Anemone taipaiensis* to provide new genome information for the phylogenetic analysis of Ranunculaceae.

The plant materials were sampled from Taibai Mountain (107.77°E, 33.95°N, 3200 m), Shaanxi Province, China. The vouchers (2017LIU065 and 2017LIU070) are deposited at the Evolutionary Botany Laboratory (EBL), Northwest University. Genome DNA was isolated with the modified CTAB method and sequenced in the Illumina HiSeq 2500 platform. Treatments of raw reads followed the previous method (Peng et al. 2019). *Trollius chinensis* (MK569501) and *Anemone raddeana* (MK569472) were used as the reference to assemble and annotate the plastomes of *Trollius farreri* and *Anemone taipaiensis*, respectively.

The chloroplast genome sequence of *Trollius farreri* (GenBank accession number MK843818) is 160,612 bp in length with a large single copy (LSC) region of 88,944 bp and a small single copy (SSC) region of 18,532 bp, separated by a pair of inverted repeat regions (IRs) of 26,568 bp. It has 131 genes, including 87 protein-coding genes, 8 rRNA, and 36 tRNA genes. The cpDNA of *Anemone taipaiensis* (GenBank accession number MK843819) is 160,214 bp in length, while the corresponding values of LSC, SSC, and IR are 80,850, 17,182, and 31,091 bp, respectively. It harbours 137 genes, 93 of protein-coding genes, 8 rRNA, and 36 tRNA genes. The two chloroplast genomes have similar GC contents (38.0% in *Trollius farreri* and 37.6% in *Anemone taipaiensis*). Twenty-four dispersal repeats and 21 tandem repeats were identified in *Trollius farreri* by using program REPuter (Kurtz et al. 2001) and Tandem Repeats Finder (Benson 1999). Much more number of repeats (80 dispersal repeats and 49 tandem repeats) was found in *Anemone taipaiensis*. Twenty-one plastomes from six tribes in Ranunculaceae were used to construct maximum-likelihood tree with 1000 bootstrap replicates. The results showed that *Trollius farreri* and *Anemone taipaiensis* were clustered with their congeneric taxa (Figure 1) and species in the same tribe grouped together. But relationships among tribes were not coincident with the previous reports (Liu et al. 2018; Zhai et al. 2019) and should be investigated further in future.

**Figure 1.**
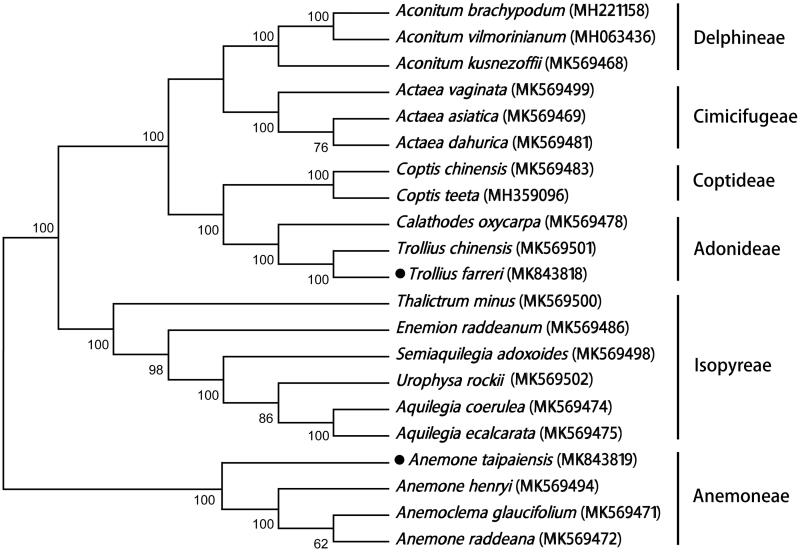
Phylogenetic tree of 21 representatives in Ranunculaceae constructed with maximum-likelihood method using the complete chloroplast genome sequences.
